# Solar Radiation Effects on Dry Matter Accumulations and Transfer in Maize

**DOI:** 10.3389/fpls.2021.727134

**Published:** 2021-09-16

**Authors:** Yunshan Yang, Xiaoxia Guo, Guangzhou Liu, Wanmao Liu, Jun Xue, Bo Ming, Ruizhi Xie, Keru Wang, Peng Hou, Shaokun Li

**Affiliations:** ^1^Key Laboratory of Crop Physiology and Ecology, Ministry of Agriculture and Rural Affairs/Institute of Crop Sciences, Chinese Academy of Agricultural Sciences, Beijing, China; ^2^The Key Laboratory of Oasis Eco-Agriculture, Xinjiang Production and Construction Corps/College of Agronomy, Shihezi University, Shihezi, China

**Keywords:** maize, solar radiation, density, dry matter accumulation and translocation, photosynthates, leaf area duration

## Abstract

Solar radiation is the energy source for crop growth, as well as for the processes of accumulation, distribution, and transfer of photosynthetic products that determine maize yield. Therefore, learning the effects of different solar radiation amounts on maize growth is especially important. The present study focused on the quantitative relationships between solar radiation amounts and dry matter accumulations and transfers in maize. Over two continuous years (2017 and 2018) of field experiments, maize hybrids XY335 and ZD958 were grown at densities of 4.5 × 10^4^ (D1), 7.5 × 10^4^ (D2), 9 × 10^4^ (D3), 10.5 × 10^4^ (D4), and 12 × 10^4^ (D5) plants/ha at Qitai Farm (89°34′E, 44°12′N), Xinjiang, China. Shading levels were 15% (S1), 30% (S2), and 50% (S3) of natural light and no shading (CK). The results showed that the yields of the commonly planted cultivars XY335 and ZD958 at S1, S2, and S3 (increasing shade treatments) were 7.3, 21.2, and 57.6% and 11.7, 31.0, and 61.8% lower than the control yields, respectively. Also, vegetative organ dry matter translocation (DMT) and its contribution to grain increased as shading levels increased under different densities. The dry matter assimilation amount after silking (AADMAS) increased as solar radiation and planting density increased. When solar radiation was <580.9 and 663.6 MJ/m^2^, for XY335 and ZD958, respectively, the increase in the AADMAS was primarily related to solar radiation amounts; and when solar radiation was higher than those amounts for those hybrids, an increase in the AADMAS was primarily related to planting density. Photosynthate accumulation is a key determinant of maize yield, and the contributions of the vegetative organs to the grain did not compensate for the reduced yield caused by insufficient light. Between the two cultivars, XY335 showed a better resistance to weak light than ZD958 did. To help guarantee a high maize yield under weak light conditions, it is imperative to select cultivars that have great stay-green and photosynthetic efficiency characteristics.

## Introduction

Food shortage has long been a worldwide problem (Jia et al., 2011), but the recent COVID-19 pandemic, beginning in early 2020, has not only seriously affected public health but has also added significant uncertainty to national and global food supplies (Balwinder et al., 2020; Lamichhane and Reay-Jones, 2021). One of the most important crops globally, maize, provides food and protein for people, as well as raw material for industrial production (Gao et al., 2017). However, maize production is vulnerable to abnormal weather conditions, such as continuous rain, wet weather, and low-light levels caused by cloud cover, and that has been exacerbated due to worldwide climate change and environmental pollution (Wu et al., 2020). Solar radiation drives crop photosynthesis and yields, as well as the formation and development of plant organs (Ding et al., 2005; Zhang et al., 2007; Dordas, 2009; Ye et al., 2020). Studies have shown that global solar radiation has been decreasing by an average of 1.4–2.7% per decade, and the effective sunlight duration decreasing by 1.28% each decade over a period of time in China (Cui et al., 2015; Ren et al., 2016). For example, in the Huang-Huai Plain region, predicted maize yields could be reduced by 3–6% by rainy weather and insufficient light during the growing period, especially given the background of global climate change (Cui et al., 2012; Ren et al., 2016; Gao et al., 2017). Therefore, exactly how solar radiation changes affect maize production must be investigated to help guarantee maize yield under future climate change scenarios.

Dry matter production, accumulation, and transportation are important factors that determine maize yield (Hou et al., 2020; Liu et al., 2020a,b), which is significantly correlated with the continuous increase of dry matter accumulation after flowering (Zhang et al., 2016). Gao et al. (2017) suggested that ~60% of the carbohydrates in maize grains come from post-flower photosynthetic products, whereas Yan et al. (2001) suggested that higher yielding cultivars have stronger post-flowering photosynthetic capacity but poor assimilate transfer to grain. Nevertheless, some studies posited that the main reason for higher maize yields is the accumulation of more dry matter at the pre-silking stage and a higher transport rate in the post-silking stage (Yang et al., 1999). Barnabás et al. (2007) demonstrated that maize grain yield is dependent on post-silking photosynthate accumulation, but the translocation of reserved carbohydrates in vegetative organs to grains cannot be ignored (Mu et al., 2010; Wang et al., 2020a; Ye et al., 2020). Maize yield may effectively be increased by increasing dry matter production capacity and then transferring as much of that accumulated dry matter to the grain as possible (Chen, 1994; Ding et al., 2005; Hou et al., 2012). Although aboveground dry matter accumulation, partitioning, and translocation have been well documented in rice (Yang et al., 1997), wheat (Dordas, 2009; Zhou et al., 2012), cotton (Ibrahim et al., 2010), and maize (Zhu et al., 2011; Pu et al., 2016), little is known about the effects of solar radiation on dry matter accumulation and translocation in maize.

Field shading, a common method used to study the effects of solar radiation on crop growth (Yang et al., 2001; Cui et al., 2015; Ren et al., 2016; Fan et al., 2018), shows how different shading periods have different effects on maize growth (Zhang et al., 2006; Cui et al., 2013a; Shi et al., 2015; Gao et al., 2017). Shading during the reproductive period of the maize decreases grain yield more than during the vegetative growth stages (Early et al., 1967; Zhang et al., 2007; Yang et al., 2019). Furthermore, different degrees of shading have different effects on maize growth and development (Cui et al., 2013a). The accumulation and distribution of dry matter in the stem, leaf, and sheath are important factors in maize grain yield (Karlen et al., 1987; Gao et al., 2017; Yang et al., 2021). Also, assimilates in the vegetative organs gradually move to the grain in the late growth stage (Yang et al., 1997; Ma et al., 2008; Gao et al., 2017). Modern maize grain yield improvements are highly dependent on increasing plant density while enabling the plants to intercept more solar radiation (Liu et al., 2017, 2021c; Hou et al., 2020), and planting density affects light quality and other environmental factors that influence the yield as well (Jin et al., 2020). Also, planting density has important effects on maize dry matter partitioning between vegetative and reproductive organs (Wei et al., 2019), as planting density increases, the numbers of vegetative organs increase while that of reproductive organs decrease (Liu et al., 2011). Previous studies have indicated that leaf area index (LAI) increases as plant density increases (Xu et al., 2017; Liu et al., 2020a), an overly high LAI may cause self-shading and has been noted for possible photosynthetic decrease and yield loss (Cui et al., 2013b; Liu et al., 2015, 2020a; Srinivasan et al., 2017), and the increase of leaf area duration (LAD) of maize was accompanied by the increase of photosynthetic rate, and finally significantly increased the total biomass (Liu et al., 2020a).

There have been many studies on shading (Andrade et al., 1993; Andrade and Ferreiro, 1996; Cerrudo et al., 2013), however, little is known about the interactive and quantitative relationships between solar radiation, planting density, and hybrids in maize. Additionally, because most of the previous studies were conducted in lower solar radiation areas in China (Jia et al., 2007; Cui et al., 2015; Ren et al., 2016), their findings were not closely connected to the actual production conditions after shading. In this study, we chose a farm in the Xinjiang region, the area with the most abundant solar radiation in China (Xue et al., 2016), and the two most widely planted maize genotypes were selected. We also established different shading and planting density treatments to re-create different solar radiation conditions so that we could study the quantitative relationships between maize dry matter accumulations and transfers and solar radiation. Our results provide a theoretical basis for cultivar breeding and improved field management as agronomists cope with climate change and dense planting.

## Materials and Methods

### Experimental Design

We conducted field experiments in 2017 and 2018 at the Qitai Farm (43°49′27″N,89°48′22″E) in Xinjiang, China. A split block design was conducted with cultivars as the main factor, planting density as the subplot factor, and shading level as the secondary subplot factor, and all plots were arranged in a completely randomized design with three replications. We used maize hybrids Xianyu 335 (XY335) and Zhengdan 958 (ZD958) in both the years because they are widely grown in China, and the plant architecture of these two hybrids was different, such as leaf length and leaf angles (Ma et al., 2014; Hou et al., 2020). The experimental plots measured 11 × 10 m and adjacent plots were separated by a 1 m wide walkway. Different environmental solar radiation conditions were created by manipulating shading and planting density. The maize was planted at five different densities: 4.5 × 10^4^ (D1), 7.5 × 10^4^ (D2), 9 × 10^4^ (D3), 10.5 × 10^4^ (D4), and 12 × 10^4^ (D5) plants/ha in 2018 and three planting densities (D2, D4, and D5) in 2017. Shading levels were 50 (S3), 30 (S2), and 15% (S1) of natural light and no shading (CK). We used nylon nets to build temporary shading sheds. The nets were 4.5 m above the ground, which were fixed in place ~1.5 m above the maize canopy in order to maintain the same microclimatic conditions except for solar radiation as in the unshaded portions of the field. The shading period began at silking and lasted until maturity. Shading nets were designed and fabricated to have different shading strengths, and the incident light quality in the maize canopy was not affected by field shading (Andrade et al., 2000; Jia et al., 2011; Yang et al., 2020).

All experimental plots were irrigated (15 mm) on the 1st day after sowing, and starting from 60 days after sowing, single water applications of 58 mm were delivered at 9–10 day intervals throughout the growing season for a total of nine applications. The total irrigation amount was ~540 mm (Zhang et al., 2017). All weeds, diseases, and pests were controlled. Base fertilizers were applied before sowing and included 150 kg/ha N from urea, 225 kg/ha P_2_O_5_ (super phosphate), and 75 kg/ha K_2_O (from potassium sulfate). To ensure a non-limiting supply of nutrients, additional urea (300 kg/ha N) was applied *via* drip irrigation in alternate irrigations during the growing season.

### Sampling and Measurement

In each plot, three adjacent plants from the same inside row were cut manually at silking and at physiological maturity. We assigned plant part categories as stalk (stalk, sheath, and tassel), leaf, cob, husk, and grain; and after harvest, the parts were oven dried (85°C) to a constant weight. At physiological maturity, a 3.3 × 5 m area [in an alternating narrow–wide (40:70 cm) row planting pattern] was manually harvested from the center of each plot and its grain weight was measured (Liu et al., 2020a). We determined grain moisture content using a PM8188 portable moisture meter (Kett Electric Laboratory, Tokyo, Japan), and grain yield and thousand kernel weights (TKW) were determined at 14% moisture content. The kernel rows per ear and kernel number per row were calculated using 10 selected ears. The kernel number per ear (KNP) was calculated as follows: KNP = kernel rows per ear × kernel number per row (Liu et al., 2019). In 2018, every 10 days after silking and until maturity, leaf area measurements [leaf length (L) and maximum leaf width (W) of all the leaves on each tagged plant) were taken from five marked, representative plants from each plot. Then leaf areas and LAIs were calculated as described by Xu et al. (2017).


(1) $$\begin{array}{l} \text{Leaf area }=\text{ L }\times \text{ W }\times \;0.75 \end{array}$$

(2) $$\text{LAI = }\frac{\text{Leaf area per plant }\times \text{ plant number per plot}}{\text{Plot area}}$$

Leaf area duration (LAD) was calculated as:


(3) $$\begin{array}{l} LAD\;=\;\frac{L1\;+\;L2}{2}\times \;\left(t1\;-\;t2\right) \end{array}$$

where L1 and L2 are the leaf area per plant at time *t*1 (maturity) and *t*2 (silking), respectively (Liu et al., 2021a).

We obtained meteorological data for the 2017 and 2018 maize growing seasons from a WatchDog 2000 Weather Station data logger (Spectrum Technologies, Inc., Washington, DC, United States) located in the experimental field (the data were recorded at hourly intervals), and the measured PAR was averaged in the wide and narrow rows at the top and the bottom of the canopies at 13:00 and 15:00 hours (Xu et al., 2017) on clear days using a SunScan (Delta-T Devices, Cambridge, United Kingdom). The total intercepted PAR was calculated according to the following formula.


(4) $$\begin{array}{l} \text{Total intercepted PAR }\left(\text{MJ}/\text{m}2\right)\;=\;\left(1-\frac{B}{A}\right)\;\times \;C\;, \end{array}$$

where *A* is PAR above the canopy, *B* is the transmitted PAR at the bottom of the canopy, and *C* is total accumulated par from silking to maturity.

In 2018, ear leaves per plot were chosen for photosynthesis measurement during the grain filling stage (20 days after silking). First, gas exchange measures were made on clear days at 13:00 and 15:00 using an LI-6400 programmable, portable open-flow gas exchange system (Li-Cor Inc., Lincoln, NE, United States). We performed light induction by keeping the leaves in the leaf chamber with the CO_2_ concentration controlled at 400 μmol CO_2_ (per mol air) and under PAR = 2,000 μmol/m^2^/s until the parameter readings were stable (Liu et al., 2020a). Dry matter translocation (DMT) of vegetative organs (stalk + leaf), contribution of pre-silking dry matter to grain (CDMG), and the amount of assimilated dry matter after silking (AADMAS) were calculated as described by Zhu et al. (2011) and all weights were measured as *t*/ha.


(5) $$\begin{array}{l} \text{DMT of vegetative organs }=\text{ Dry matter weight at silking}\\ \;-\text{ Dry matter weight at maturity} \end{array}$$

(6) $$\begin{array}{l} \text{ CDMG of the vegetative organ }(\%)\\ \text{= }\frac{\text{DMT of the vegetative organ}}{\text{Kernel dry matter weight at maturity }}\;\times \text{ 100} \end{array}$$

(7) $$\begin{array}{l} \text{AADMAS }=\text{ Dry matter weight of grain at maturity}\\ \;-\text{DMT of vegetative organs} \end{array}$$

### Statistical Analysis

Statistical calculations were performed and charts generated in Excel 2016 (Microsoft, Redmond, WA, United States) and Origin 2018 (OriginLab, Northampton, MA, United States). SPSS ver. 21.0 (IBM SPSS, Chicago, IL, United States) was used to conduct one-way ANOVA followed by Duncan's multiple range tests at *P* < 0.05 to test the differences between different treatments in the two study years. Treatment effects and interaction between treatments were analyzed by ANOVA using mixed models. Residuals were analyzed to corroborate the assumptions of the ANOVA. For all of the dependent variables analyzed, year, cultivar, density, and shading level were considered as fixed factors.

## Results

### Different Shading Levels Affect Maize Yield, Yield Components, and Dry Weight of Organs Under Different Density Conditions

Shading affected maize yield, the decrease rate of yield was in the order S3 > S2 > S1, compared with CK (Table 1). Over the 2 years of the experimental period, the mean yields of five planting densities of XY335 were >ZD958; and compared with CK, yields of XY335 decreased < ZD958 after shading. Averaging all planting densities (D1, D2, D3, D4, and D5), and over both the study years, the yields of XY335 and ZD958 at S1, S2, and S3 were 7.3, 21.2, and 57.6%, and 11.7, 31.0, and 61.8% lower than CK, respectively. Also, the dry matter weight of vegetative organs at maturity were 8.7, 8.9, and 18.2%, and 4.5, 10.7, and 20.2% lower than CK, respectively (Table 1). Averaging all shading treatments, the yields of XY335 and ZD958 at D1, D2, D3, D4, and D5 were 21.9, 21.1, 30.0, 31.6, and 35.1% and 13.3, 18.2, 42.8, 40.4, and 39.9%, the vegetative organ dry matter weights were 18.6, 17.0, 17.8, 21.6, and 12.8% and 16.2, 13.4, 16.3, 14.0, and 8.2% lower than CK, respectively. The reduction of ear density, KNP, and TKW significantly increased with the increase of shade level (Table 2). The main effect of shading treatment on maize yield components was the decrease in KNP and TKW and therefore the shading mainly affected grain formation and filling after silking. For the cultivars, the KNP and TKW of XY335 were higher than that of ZD958.

**Table 1:** Effects of different shading levels (CK, natural light; S1, 15% natural light; S2, 30% natural light; S3, 50% natural light) and planting densities (D1, 4.5 × 10^4^ plants ha^−1^; D2, 7.5 × 10^4^ plants ha^−1^; D3, 9 × 10^4^ plants ha^−1^; D4: 10.5 × 10^4^ plants ha^−1^; D5, 12 × 10^4^ plants ha^−1^) on maize grain yields and dry weights of plant organs (dry matter of vegetative organs at silking [VS] and at maturity [VM]) in 2017 (Y1) and 2018 (Y2).

**XY335**	**ZD958**
**Treatment**	**Yield (t ha^**−1**^)**	**VS (t ha^**−1**^)**	**VM (t ha^**−1**^)**	**Yield (t ha^**−1**^)**	**VS (t ha^**−1**^)**	**VM (t ha^**−1**^)**
Y1D2CK	18.46 a	11.3 a	14.9 a	17.15 a	10.3 a	11.8 a
Y1D2S1	18.55 a	11.3 a	12.3 a	16.92 a	10.3 a	11.2 a
Y1D2S2	17.02 a	11.3 a	13.7 a	13.90 b	10.3 a	11.2 a
Y1D2S3	12.42 b	11.3 a	11.9 a	8.77 c	10.3 a	9.3 b
Y1D4CK	19.92 a	14.8 a	17.0 a	19.29 a	14.7 a	13.5 a
Y1D4S1	18.16 ab	14.8 a	13.5 b	16.66 ab	14.7 a	13.5 a
Y1D4S2	16.20 b	14.8 a	13.7 b	13.87 b	14.7 a	13.1 a
Y1D4S3	11.13 c	14.8 a	13.9 b	8.46 c	14.7 a	11.0 b
Y1D5CK	21.76 a	16.3 a	16.4 a	20.13 a	15.1 a	16.1 a
Y1D5S1	19.93 ab	16.3 a	15 b	17.34 b	15.1 a	15.3 a
Y1D5S2	16.99 b	16.3 a	13.3 c	14.14 c	15.1 a	15.0 a
Y1D5S3	10.29 c	16.3 a	12.8 c	7.55 d	15.1 a	15.0 b
Y2D1CK	18.39 a	7.9 a	10.9 a	14.74 a	7.5 a	9.7 a
Y2D1S1	17.12 a	7.9 a	9.1 ab	15.14 a	7.5 a	8.8 a
Y2D1S2	17.13 a	7.9 a	9.6 ab	15.20 a	7.5 a	8.6 a
Y2D1S3	8.84 b	7.9 a	7.9 b	8.01 b	7.5 a	7.1 b
Y2D2CK	19.29 a	12.0 a	11.3 a	17.61 a	11.6 a	12.1 a
Y2D2S1	16.85 a	12.0 a	11.7 a	16.63 a	11.6 a	11.0 a
Y2D2S2	16.76 a	12.0 a	12.6 a	10.97 b	11.6 a	10.6 a
Y2D2S3	7.96 b	12.0 a	11.4 a	7.28 c	11.6 a	8.2 b
Y2D3CK	19.21 a	14.0 a	12.8 a	17.77 a	12.7 a	12.1 a
Y2D3S1	17.69 b	14.0 a	10.7 a	14.98 b	12.7 a	11.6 a
Y2D3S2	15.05 c	14.0 a	10.8 a	10.12 c	12.7 a	9.5 b
Y2D3S3	7.58 d	14.0 a	10.2 a	5.40 d	12.7 a	9.3 b
Y2D4CK	20.06 a	13.6 a	12.1 a	18.16 a	16.2 a	14.3 a
Y2D4S1	17.98 a	13.6 a	13.2 a	13.89 b	16.2 a	12.8 ab
Y2D4S2	14.25 b	13.6 a	12.9 a	9.27 c	16.2 a	11.7 b
Y2D4S3	4.26 c	13.6 a	9.2 b	5.04 d	16.2 a	11.1 b
Y2D5CK	21.27 a	13.5 a	12.8 ab	18.99 a	15.0 a	10.4 ab
Y2D5S1	20.55 a	13.5 a	13.3 a	15.48 b	15.0 a	11.3 a
Y2D5S2	11.41 b	13.5 a	11.9 ab	11.74 c	15.0 a	9.7 bc
Y2D5S3	4.70 c	13.5 a	11.3 b	4.40 d	15.0 a	9.0 c

*Different lowercase letters indicate significant differences between treatments at P < 0.05*.

*The 2017 yield data was published and cited in Yang et al. (2019)*.

**Table 2:** Yield components of maize under different shading levels and planting densities.

**Treatment**	**XY335**	**ZD958**
	**Ear density (10^**3**^ ha^**−1**^)**	**KNP**	**TKW (g)**	**Ear density(10^**3**^ ha^**−1**^)**	**KNP**	**TKW (g)**
Y1D2CK	80 a	619 a	401.94 a	110 a	527 a	381.61 a
Y1D2S1	85 a	602 a	407.05 a	109 a	509 ab	341.18 b
Y1D2S2	88 a	636 a	395.36 a	97 ab	477 b	356.88 b
Y1D2S3	79 a	179 b	194.97 b	86 b	314 c	110.77 c
Y1D4CK	106 a	554 a	411.27 a	118 a	576 a	364.90 a
Y1D4S1	107 a	496 b	384.37 b	116 a	513 b	346.15 a
Y1D4S2	95 b	495 b	351.63 c	101 b	444 c	324.08 b
Y1D4S3	84 c	113 c	192.55 d	91 c	211 d	120.97 c
Y1D5CK	121 a	525 a	374.10 a	135 a	469 a	383.35 a
Y1D5S1	122 a	464 b	384.20 a	122 b	470 a	326.92 b
Y1D5S2	106 a	428 b	350.95 a	113 c	453 b	326.59 b
Y1D5S3	110 a	93 c	185.57 b	92 d	147 c	222.58 c
Y2D1CK	75 a	639 a	430.99 a	67 a	611 a	452.63 a
Y2D1S1	74 a	643 a	428.66 ab	66 a	602 a	432.69 ab
Y2D1S2	67 a	642 a	404.82 b	63 a	601 a	421.69 b
Y2D1S3	46 b	521 b	372.67 c	43 b	537 b	362.93 c
Y2D2CK	78 a	615 a	424.43 a	74 ab	604 ab	398.05 a
Y2D2S1	70 a	621 a	405.82 a	73 ab	616 a	389.39 a
Y2D2S2	73 a	563 a	398.83 ab	77 a	544 b	340.76 b
Y2D2S3	70 a	323 b	376.13 c	64 b	414 c	312.94 c
Y2D3CK	84 ab	577 a	420.73 a	90 a	576 a	369.37 a
Y2D3S1	89 a	596 a	395.79 b	86 a	581 a	341.73 b
Y2D3S2	74 b	526 a	387.74 b	89 a	525 a	319.84 bc
Y2D3S3	74 b	266 b	371.57 c	72 b	262 b	300.10 c
Y2D4CK	100 a	577 a	402.60 a	93 a	564 a	378.72 a
Y2D4S1	94 a	569 a	376.37 a	93 a	515 a	363.60 a
Y2D4S2	86 ab	454 b	377.54 a	94 a	548 a	342.60 ab
Y2D4S3	72 b	372 c	371.86 a	65 b	307 b	300.86 b
Y2D5CK	108 a	544 a	405.07 a	103 a	535 a	353.48 a
Y2D5S1	105 a	507 ab	383.54 ab	109 a	463 b	315.52 ab
Y2D5S2	93 ab	399 b	369.24 b	99 a	408 c	322.27 ab
Y2D5S3	73 b	248 c	366.41 b	81 b	221 d	262.83 b

*Different lowercase letters indicate significant differences between treatments at P < 0.05. KNP, kernel number per ear. TKW, thousand-kernel weight. See Table 1 for planting density and shading treatment definitions*.

### Effects of Different Shading Levels on DMT of Vegetative Organs and CDMG Under Different Density Conditions

**Figure 1:**
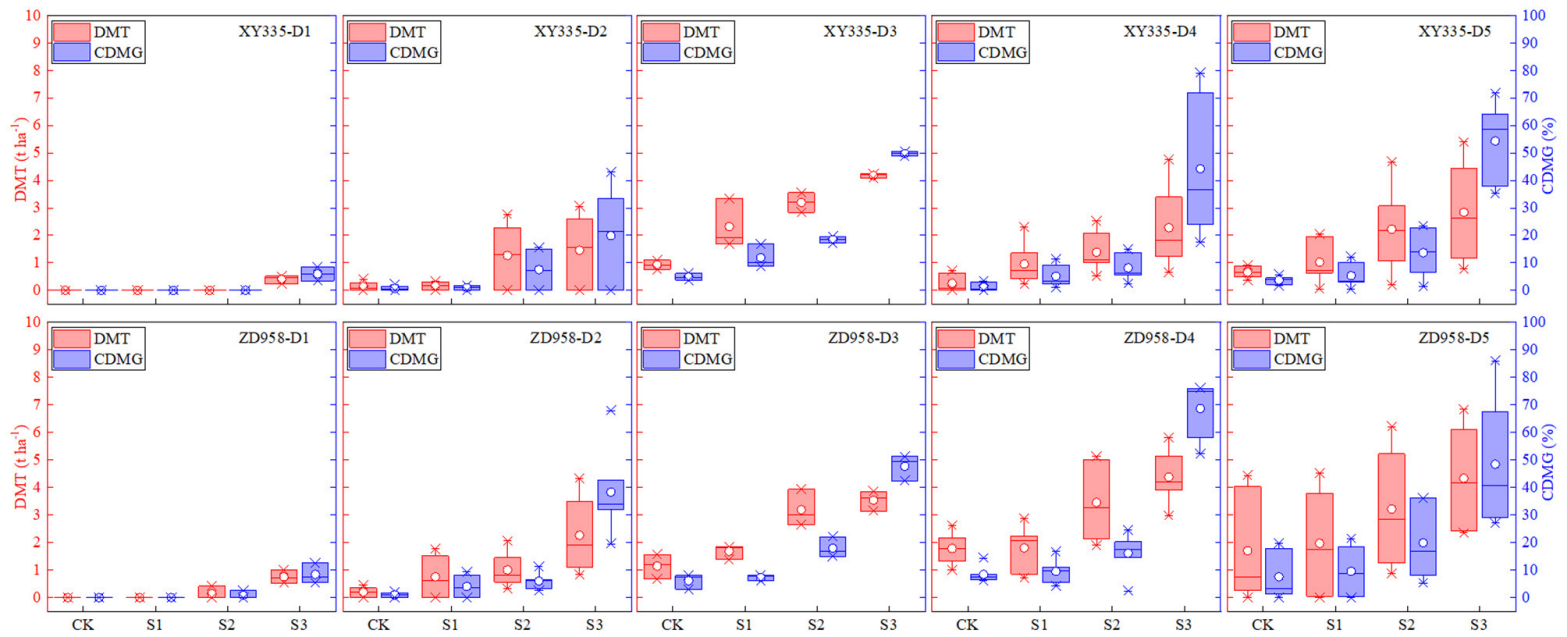
Effects of different shading levels (CK, natural light; S1, 15% natural light; S2, 30% natural light, S3, 50% natural light) on vegetative organ dry matter translocation (DMT) and pre-silking dry matter contributions to grain (CDMG) under different planting densities (D1, 4.5 × 10^4^ plants/ha; D2, 7.5 × 10^4^ plants/ha; D3, 9 × 10^4^ plants/ha; D4, 10.5 × 10^4^ plants/ha; D5, 12 × 10^4^ plants/ha). Maize hybrids: XY335, Xianyu 335, and ZD958, Zhengdan 958. Boxes, 25th and 75th percentiles; interior circles and bars, mean and median, respectively; bars, minimum and maximum values.

Both vegetative organ DMT and pre-silking CDMG increased as shading level increased under different densities (Figure 1). These results showed that over the 2 years and five planting densities, the mean DMTs in CK, S1, S2, and S3 were 0.68, 1.07, 1.91, and 2.01 *t*/ha, while the mean CDMGs were 3.52, 5.54, 11.28, and 41.25% (15.4% total), respectively. Shading increased DMT by averages of 56.5, 179.4, and 196.1%, and increased CDMG by averages of 0.6, 2.23, and 10.45% in S1, S2, and S3, respectively, compared with those measures in CK. We also showed that the 2-year DMT and CDMG averages of all shading treatments (CK, S1, S2, and S3) for D1, D2, D3, D4, and D5 were 0.16, 0.91, 2.52, 2.04, and 1.47 *t*/ha and 1.92, 9.83, 20.52, 20.21, and 20.33%, respectively. DMT and CDMG rates increased more for XY335 than for ZD958, thus indicating that XY335 transferred more photosynthetic products to grain than ZD958 under low–solar radiation stress. The XY335 and ZD958 DMTs in CK were 0.40 and 0.97 *t*/ha, respectively, but those measures increased significantly in S1, S2, and S3: by 123.9, 304.8, and 458.4% for XY335 and by 28.6, 127.6, and 215.9% for ZD958. Although DMTs increased with the increase of shade levels, the amplitude of the changes between them was not proportional. Likewise, the mean CDMG over both years and all planting densities of XY335 and of ZD958 under S1, S2, and S3 increased significantly (by 121.7, 354.2, and 1549.8% for XY335 and by 31.7, 162.9, and 813.9% for ZD958) compared with those measures for CK (2.11 and 4.63% for XY335 and ZD958, respectively).

### Quantitative Relationships Between AADMAS and Planting Densities Under Different Solar Radiation Levels

**Figure 2:**
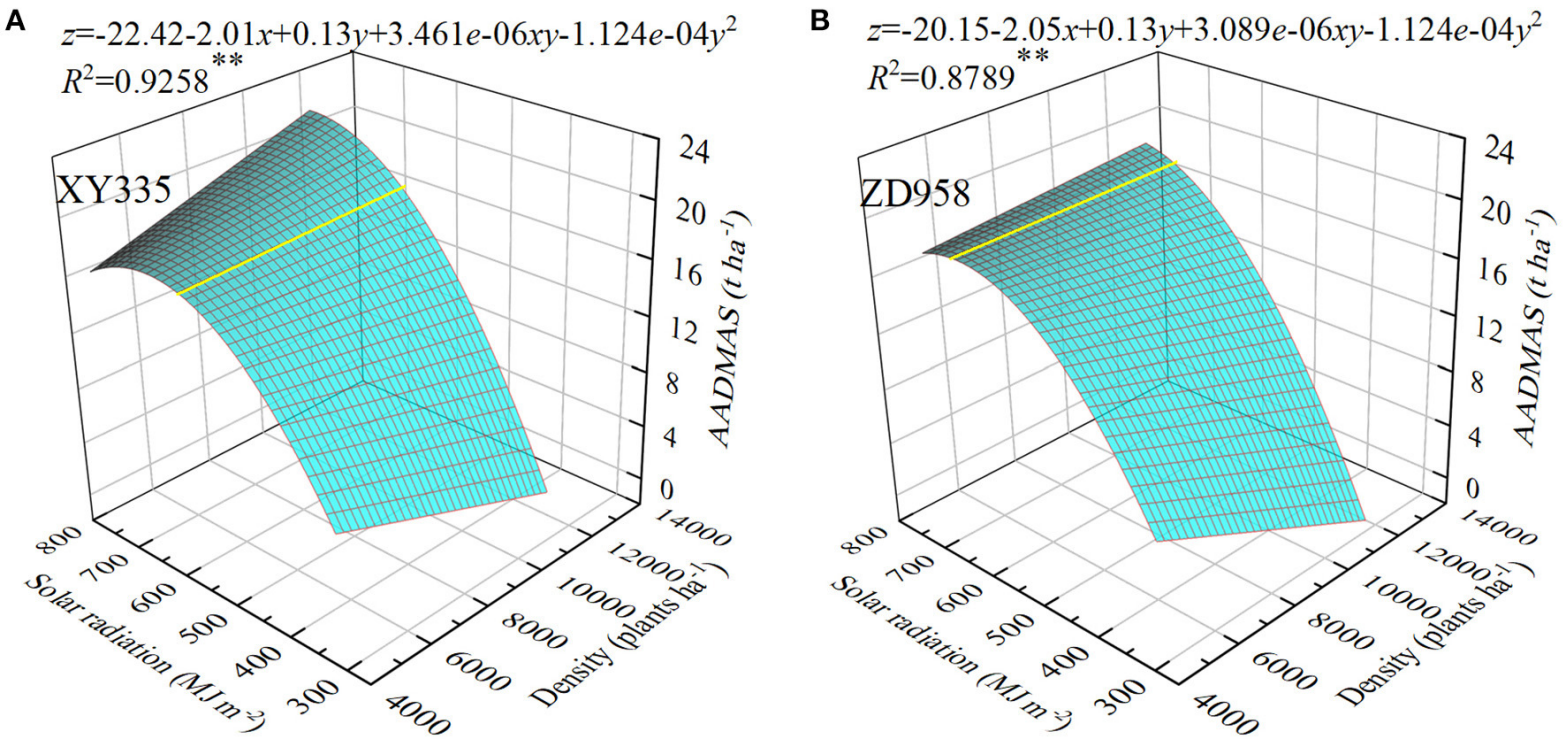
Relationships between assimilation amount of dry matter after silking (AADMAS), planting density, and different totals of accumulated photosynthetically active radiation from the silking to the maturity stages in two maize hybrids (XY335 and ZD985). *x* is planting density, *y* is solar radiation, *z* is AADMAS. **P ≤ 0.01.

As solar radiation increased so did AADMAS, which also decreased as planting density increased when solar radiation was low, but increased at the same planting densities when radiation was high (Figures 2A,B). For three-dimensional analysis, we used multiple linear regression to evaluate the interaction effects between planting density and solar radiation on AADMAS in XY335 and ZD958. Combined planting density and solar radiation explained 93% and 88% of the variations in AADMAS for XY335 and ZD958, respectively. When the solar radiation was <580.9 and 663.6 MJ/m^2^, for XY335 and ZD958, respectively, increases in AADMAS were primarily related to the amount of solar radiation. When the solar radiation was higher than 580.9 and 663.6 MJ/m^2^ for XY335 and ZD958, respectively, increases in AADMAS were primarily related to planting density. The XY335 and ZD958 AADMAS of CK were 19.7 and 19.2 *t*/ha, respectively. AADMAS decreased significantly in S1, S2, and S3 by 8.7, 22.7, and 80.3% for XY335 and by 7.8, 27.3, and 81.8% for ZD958. Averaging all shading leves and over both the study years, the AADMAS of XY335 and ZD958 D1, D2, D3, D4, and D5 were 26.0, 32.5, 36.6, 39.8, and 45.2%, and 26.6, 32.8, 35.4, 46.9, and 45.1% lower than CK, respectively. The fluctuations of AADMAS and shading level were not synchronous, and which also increased as planting density increased.

### Influences of Shading on Photosynthetic Characteristics and LAD of Different Maize Cultivars

**Figure 3:**
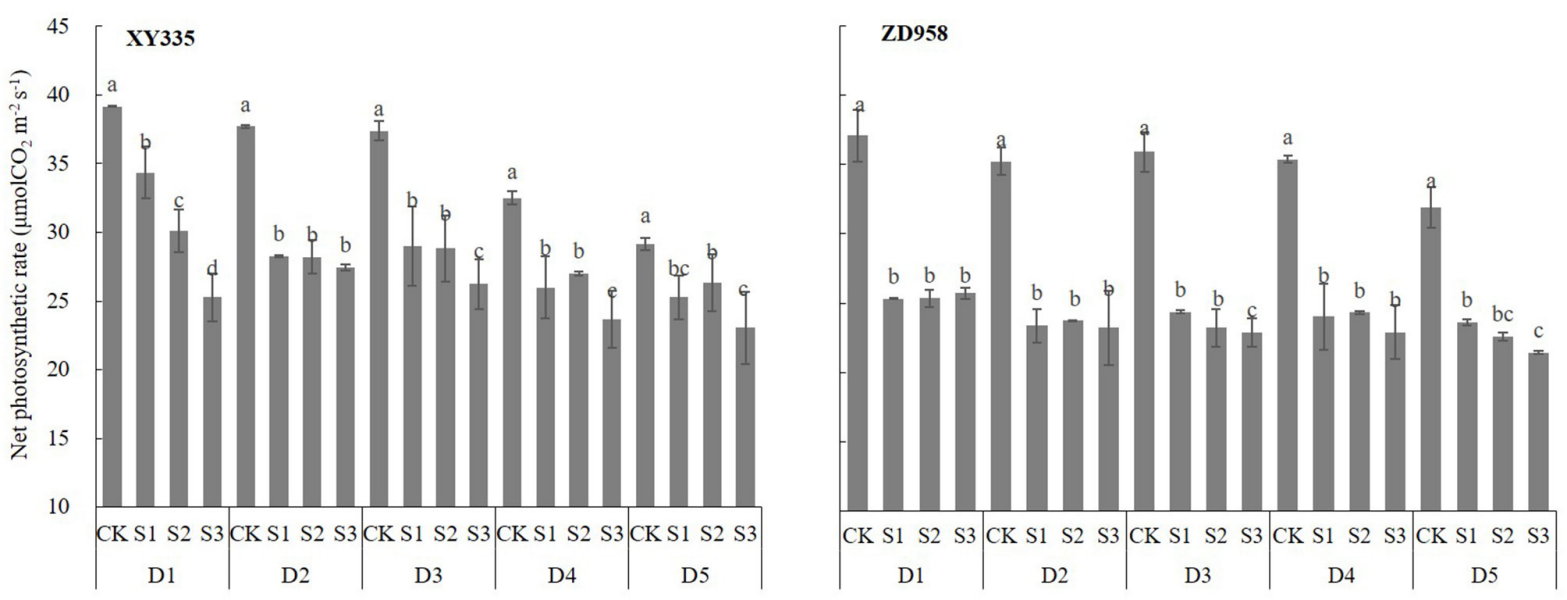
Ear leaf photosynthetic rate (*Pn*) at the grain-filling stage of maize cultivars XY335 and ZD958 under low-light stress in 2018. See Figure 1 for planting density and shading treatment definitions. Different lowercase letters of the same cultivar above the columns show significant differences between each shading treatment for each planting density at *P* < 0.05.

All ear leaf photosynthetic rates (*Pn*) changed significantly after shading (Figure 3) and they decreased as shading levels and planting densities increased (*Pn* of ZD958 was not decreased with increase in plant densities). As shown in Figure 3, *Pn*s were greater for XY335 than for ZD958, as the *Pn* under S1, S2, and S3 decreased significantly by 18.6, 19.54, and 28.1%, for XY335 and by 31.11, 32, and 33.82% for ZD958. This indicated that the net leaf *Pn* decreased as shading increased, and since the ratio of the decrease of XY335 was lower than that of ZD958, XY335 had better photosynthetic characteristics than did ZD958.

**Figure 4:**
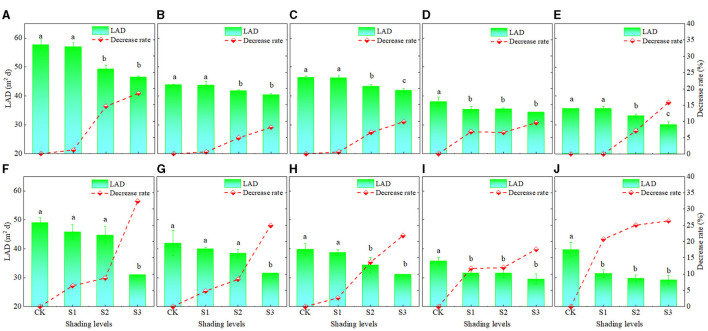
The leaf area duration (LAD) and after silking under different shading levels and planting densities treatments and the decrease rate in LAD under each shading treatment compared to the CK. **(A–E)** show the cultivar XY335 results at densities D1, D2, D3, D4, and D5, respectively, and **(F–J)** show the ZD958 results at the same densities. See Figure 1 for planting density and shading treatment definitions. Different lowercase letters of the same cultivar above the columns show significant differences between each shading treatment for each planting density at *P* < 0.05.

From silking to maturity, the LAD gradually decreased (Figure 4). Under CK, S1, S2, and S3, the LADs of XY335 were 44.3, 43.6, 40.6, and 38.6 m^2^/day, respectively, and they were 52.6, 42.4, 44.5, 35.8, and 33.7 m^2^/day, under D1, D2, D3, D4, and D5, respectively (Figures 4A–E). Under the CK, S1, S2, and S3, the LADs of ZD958 were 41.2, 37.5, 35.7, and 30.5 m^2^/day, respectively, and were 42.6, 38.0, 36.0, 32.1, and 32.5 m^2^/day, under D1, D2, D3, D4, and D5, respectively (Figures 4F–J). As shown in Figure 4, LADs were greater for XY335 than for ZD958, as the LAD under S1, S2, and S3 decreased significantly by 1.8, 7.9, and 12.4%, for XY335 and by 9.3, 13.6, and 24.6% for ZD958. This indicated that LAD decreased as shading increased. Since the decrease rate of XY335 was lower than that of ZD958, XY335 had better leaves anti-aging ability than ZD958 (Figure 4).

### Relationships Between DMT and Accumulation and Leaf *Pn*, LAD, and Their Correlations With the Yield

**Figure 5:**
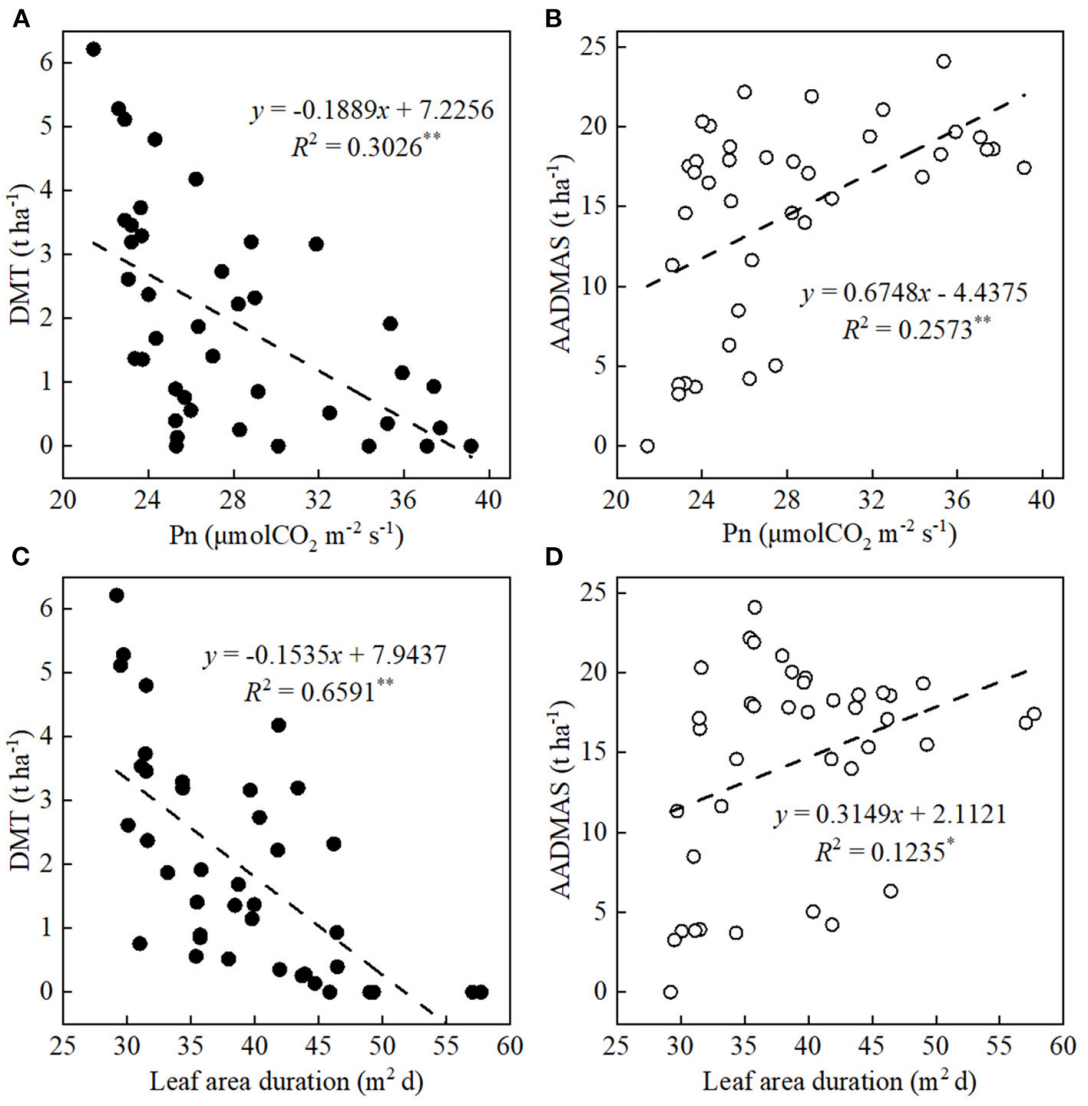
Relationships between ear leaf photosynthetic rates (*Pn*) at the grain-filling stage and LAD with DMT and AADMAS. DMT and AADMAS are indicated by solid circles and empty circles, respectively. *P ≤ 0.05, **P < 0.01.

DMT and AADMAS were significantly affected by both the *Pn*s and LAD (Figures 5A–D). First, leaf *Pn* and LAD were significantly negatively correlated with DMT and positively correlated with AADMAS, Specifically, when the *Pn* increased by 1 μmol CO_2_/m^2^/s, DMT decreased by 0.19 *t*/ha and AADMAS increased by 0.68 *t*/ha. Also, when the decreases in LAD increased by 1 m^2^/day, DMT decreased by 0.15 *t*/ha and AADMAS increased by 0.31 *t*/ha.

**Figure 6:**
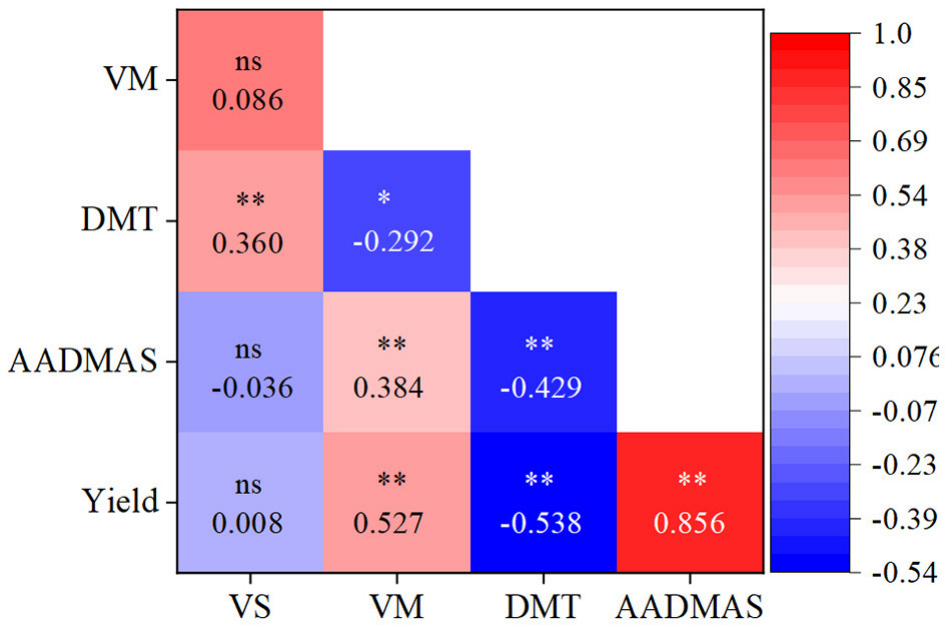
Relationships of yield, vegetative organ dry matter at silking (VS) and at maturity (VM), and AADMAS and DMT, across all treatments, including different shading levels, cultivars, and planting densities. *P < 0.05; **P < 0.01; ns, not significant.

As shown in Table 3, vegetative organ dry matter at silking (VS), at maturity (VM), ear density, and TKW were significantly affected by the interaction of Y × C; VS, VM, and TKW were significantly affected by the interaction of Y × D; yield, KNP, and TKW were significantly affected by the interaction of Y × S; VS, ear density, and TKW were significantly affected by the interaction of C × D; yield, ear density, KNP, and TKW were significantly affected by the interaction of C × S and D × S (D × S was not significant for TKW); yield, VS, VM, ear density, KNP, and TKW were significantly affected by the interaction of Y × C × D and Y × C × S (Y × C × D was not significant for yield and Y × C × S was not significant for VS); the interaction of Y × D × S was significant for TKW, the interaction of C × D × S was significant for yield and KNP, and the interaction of Y × C × D × S was significant for VM and TKW. VS and VM, as well as the AADMAS were significantly positively correlated with the yield (Figure 6). However, DMT was significantly negatively correlated with the yield.

**Table 3:** ANOVA analysis for the effects of year, cultivar, planting density and shading level on the grain yields, yield components (KNP, kernel number per ear. TKW, thousand-kernel weight) and dry weights of plant organs (dry matter of vegetative organs at silking [VS] and at maturity [VM]).

**Source**	**Yield**	**VS**	**VM**	**Ear density**	**KNP**	**TKW**
Year (Y)	**	ns	**	**	**	**
Cultivar (C)	**	ns	**	**	ns	**
Density (D)	*	**	**	**	**	**
Shading level (S)	**	ns	**	**	**	**
Y × C	ns	**	*	**	ns	**
Y × D	ns	**	**	ns	ns	**
Y × S	**	ns	ns	ns	**	**
C × D	ns	**	ns	*	ns	**
C × S	*	ns	ns	*	**	**
D × S	**	ns	ns	**	**	ns
Y × C × D	ns	**	*	**	**	**
Y × C × S	**	ns	*	**	**	**
Y × D × S	ns	ns	ns	ns	ns	**
C × D × S	*	ns	ns	ns	**	ns
Y × C × D × S	ns	ns	*	ns	ns	**

* and ** indicate significant differences at P < 0.05 and P < 0.01 probability levels, ns indicates no significance, respectively.

## Discussion

As a primary environmental factor of crop growth, light intensity importantly influences maize yield (Jia et al., 2011; Shi et al., 2013; Ren et al., 2016; Hou et al., 2021). Indeed, we found that both dry matter weight and yield under our S1, S2, and S3 treatments were lower than those measures in CK, a result that has been found in other maize shading studies (Shi et al., 2015; Guo et al., 2020; Yang et al., 2021). Also, maize grain yield is dependent on post-silking photosynthate accumulation and on the translocation of the reserved carbohydrates in vegetative organs (Barnabás et al., 2007; Wang et al., 2020a). We found that DMT and CDMG increased as shading levels increased and differed under different planting densities. Those results confirm those of Wang et al. (2020a) who reported that the translocation of pre-silking assimilates in vegetative organs increased under shading (Wang et al., 2020a). In our study, DMT increased as solar radiation decreased (Figure 1), and it was significantly negatively correlated with AADMAS and yield (Figure 6), thus suggesting that vegetative organ dry matter transportation to the grain could not compensate for the yield loss received by AADMAS under insufficient light environments. DMT and AADMAS reduction due to shading does not correspond to the magnitude of radiation reduction. The reason for this phenomenon may be that compensatory photosynthesis occurred under mild low-light conditions, while more photosynthates were used for respiration under severe low-light conditions. Previous studies observed a similar result when grain dry matter that had been transferred from other organs was mainly from stems and leaves, but that amount was not large (Liang et al., 2015); and, during shading, it could not make up for the reduced post-silking biomass accumulation, thus resulting in lower yields (Mu et al., 2010; Wang et al., 2020a).

Modern maize grain yield improvement is highly dependent on increased plant density that intercepts more solar radiation than lower densities do (Antonietta et al., 2014; Liu et al., 2017; Hou et al., 2020). Previous studies have indicated that the ratio of transfer and contribution of dry matter in the stem increased when plant density increased, but that measure in leaves was the opposite (Han et al., 2008). Our results indicated that no such transfer occurred in CK, S1, and S2, whereas that transfer and contribution did occur in S3 planted at the lowest density (D1). This suggests that photosynthetic productivity after silking could supply yield formation demands and that transfer under low-density conditions is not needed. Furthermore, as density increased, transfer occurred in the shading treatments. Also, the higher the planting density the greater transfer need, thus indicating that the photosynthate produced under high density and weak light could not satisfy yield formation (Figure 1). In support of several studies (Cui et al., 2013a; Liu et al., 2021b), our results suggest that solar radiation intensity is the limiting factor for AADMAS, and that given sufficient light radiation, increased planting density fosters increased AADMAS and thus effectively increases yield (Yang et al., 2019) (Figure 2).

Photosynthesis, the main physiological process that drives plant growth, is very sensitive to light changes (Fan et al., 2018; Wu et al., 2020). Dry-matter production, especially post-silking dry matter accumulation, is closely related to photosynthetic capacity (Liu et al., 2020a). As the main photosynthetic organs, leaves (Chen, 1994; Ye et al., 2020) provide assimilates for grain development and directly affect the final yield (Tollenaar and Daynard, 1982; Barnabás et al., 2007; Zhang et al., 2007; Mu et al., 2010). Previous studies have shown that since shading likely hinders leaf photoprotective mechanisms and chlorophyll fluorescence properties, the result is decreased net photosynthetic capacity (Cui et al., 2013b; Gao et al., 2017). Decreased photosynthetic capacity was likely due to leaf senescence (Ye et al., 2020). We found that, for the physiological traits, the LAD decreased as shading increased (Figure 4), and that was accompanied by a decreasing net *Pn*, likely a consequence of leaf senescence. Based on previous research (Qian et al., 2021), the translocation of reserved carbohydrates in vegetative organs to grains was one of the important factors that determined maize yield. If the transport exceeded 20%, it would cause early senescence of maize leaves (Qian et al., 2021). However, in the present study, the average transport (CDMG) of both tested cultivars was 15.4% (Figure 1). As shown in Figures 5 and 6, LAD was significantly negatively correlated with DMT and positively correlated with AADMAS. It meant that in the shortage of light resources condition, the sink required more transport of nutrients from the vegetative organs which would deprive the strength of leaf photosynthetic capacity and affect the production of dry matter. This might be one reason for early senescence of leaves under low light conditions in this study. On the other hand, the main resource for grain yield was still from photosynthetic products formed after silking.

Differences in yield and photosynthate accumulation and translocation under light intensity changes vary among maize cultivars (Liang et al., 2015; Wang et al., 2020a). In comparing those differences in cultivars XY335 and ZD958, we found that the average yield of XY335 was higher than that of ZD958. The sink characters of KNP and TKW of XY335 were higher than that of ZD958, and DMT and CDMG rates increased more for XY335 than for ZD958, thus indicating that XY335 transferred more photosynthetic products to grain than ZD958. This indicated that the sink capacity of XY335 was better than that of ZD958 under low–solar radiation stress, and that may promote increased DMT in XY335 (Borrás et al., 2004). Also, the photosynthetic substances produced by ZD958 were used mostly for vegetative organ growth. Comparing the results of the quantitative relationship of the two cultivars (Figure 2), the corresponding demarcation value for XY335 was smaller than that for ZD958, which indicated that XY335 had better resistance than ZD958 to weak light. Additionally, under light stress, the range of *Pn* decrease in XY335was lower than that in ZD958, and the LAD decreased rate of ZD958was faster than that of XY335. XY335 has a greater leaf source duration and dry matter production capacity than ZD958. Hou et al. (2020) reported that XY335 had a compact canopy and became more compact at the high density. Other studies showed that the optimal spatial distribution of leaves contributed to delayed leaf senescence and intercept more solar radiation to improve the photosynthetic rate and promote the production potentials of maize at high planting density (Bai et al., 2020; Liu et al., 2021a). However, Antonietta et al. (2014) reported that delayed leaf senescence did not increase yields under high planting density, this may be due to different maize hybrids. So, in this study, XY335 was better able to adapt to weak light, have anti-aging ability, and maintain a higher photosynthetic ability compared to ZD958. Consequently, those superior low-light abilities may lower yield loss caused by light deficiency (Wang et al., 2020b; Wu et al., 2020).

## Conclusions

This study of maize cultivars XY335 and ZD958 determined the differences in DMT contributions under different shading levels and planting densities, and the quantitative relationship between solar radiation, density, and the accumulation of dry matter. In conclusion, shading significantly reduced the *Pn* and LAD, which consequently reduced the amount of dry matter assimilated and thus lowered maize yield. Maize hybrid XY335 was better able to adapt to weak light, maintain a higher photosynthetic and anti-aging ability compared with the cultivar ZD958. These findings show the importance of selecting maize cultivars that have strong stay-green abilities that can guarantee good grain yields even under weak light conditions.

## Data Availability Statement

The original contributions presented in the study are included in the article/supplementary material, further inquiries can be directed to the corresponding authors.

## Author Contributions

YY, PH, and SL designed the study. YY, XG, GL, and WL performed the study. YY, XG, GL, WL, JX, BM, RX, KW, PH, and SL analyzed data and performed the statistical analyses. YY wrote the paper. All authors contributed to the article and approved the submitted version.

## Conflict of Interest

The authors declare that the research was conducted in the absence of any commercial or financial relationships that could be construed as a potential conflict of interest.

## Publisher's Note

All claims expressed in this article are solely those of the authors and do not necessarily represent those of their affiliated organizations, or those of the publisher, the editors and the reviewers. Any product that may be evaluated in this article, or claim that may be made by its manufacturer, is not guaranteed or endorsed by the publisher.
